# Supplementary material for: 89Zr-Immuno-PET with Immune Checkpoint Inhibitors: Measuring Target Engagement in Healthy Organs
Source: Cancers (Basel). 2023 Nov 23;15(23):5546. doi: 10.3390/cancers15235546 (PMC10705667; doi:10.3390/cancers15235546)
Supplement: Supplementary file 1 [file cancers-15-05546-s001.zip › cancers-2641242-supplementary.pdf]

cancers

MDPI

Supplementary Materials

Table S1. RNA sequencing data expressed as normalised tags per million (nTPM) for the five different [^{89}Zr]Zr-mAbs and four different tissues obtained from the Human Protein Atlas [Uhlen et al., 2010].

^{89}Zr-Immuno-PET Tracer	Target	Spleen	Bone Marrow	Kidneys	Brain
[^{89}Zr]Zr-BI 754111	LAG-3	23.6	1.5	1.0	0.2–0.7
[^{89}Zr]Zr-durvalumab	PD-L1	10.2	1.9	2.6	0.6–1.9
[^{89}Zr]Zr-nivolumab	PD-1	7.0	0.9	0.7	0.0–0.4
[^{89}Zr]Zr-pembrolizumab	PD-1	7.0	0.9	0.7	0.0–0.4
[^{89}Zr]Zr-ipilimumab	CTLA-4	3.9	6.1	0.6	0.4–1.3

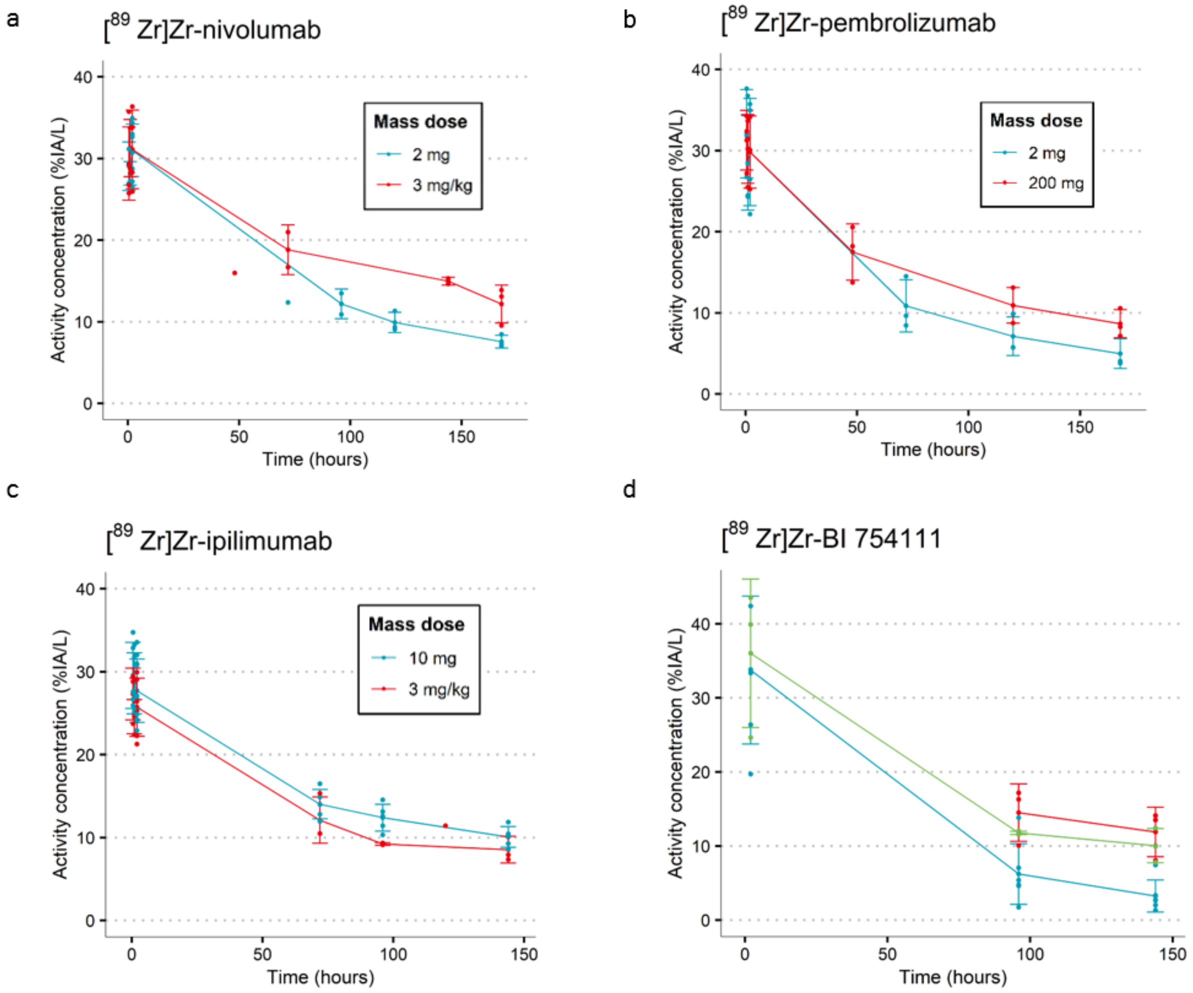

Figure S1. Plasma activity concentrations over time for two or three doses of [^{89}Zr]Zr-nivolumab (**a**), [^{89}Zr]Zr-pembrolizumab (**b**), [^{89}Zr]Zr-ipilimumab (**c**) and [^{89}Zr]Zr-BI 754111 (**d**). For time points with 2 or more observations a mean %IA/L with SD is shown. Every dot represents one observation.

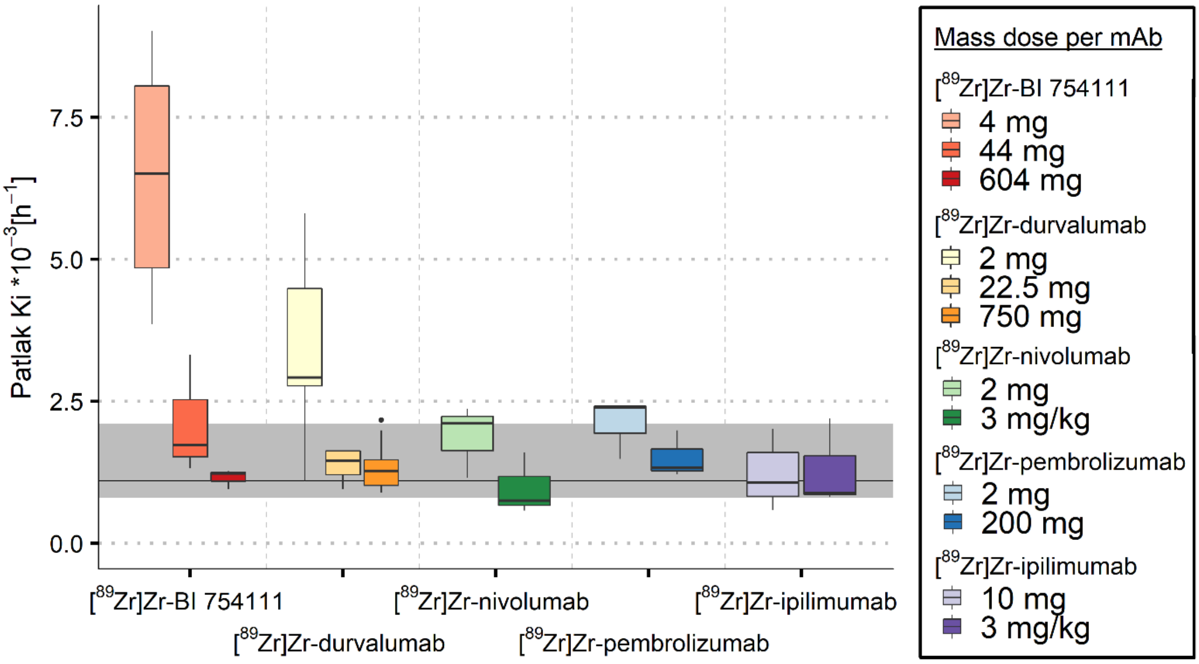

Figure S2. Irreversible uptake in liver quantified with K_i values as a function of mass dose for the five [^{89}Zr]Zr-mAbs investigated. Ns-baseline K_i median values and interquartile range for liver are indicated by the black line and grey area, respectively. K_i values decreased with increasing mass dose for all [^{89}Zr]Zr-mAbs, except for [^{89}Zr]Zr-ipilimumab. K_i values were comparable to ns-baseline values for all mass doses, except for the low mass doses of [^{89}Zr]Zr-BI 754111 and [^{89}Zr]Zr-durvalumab.

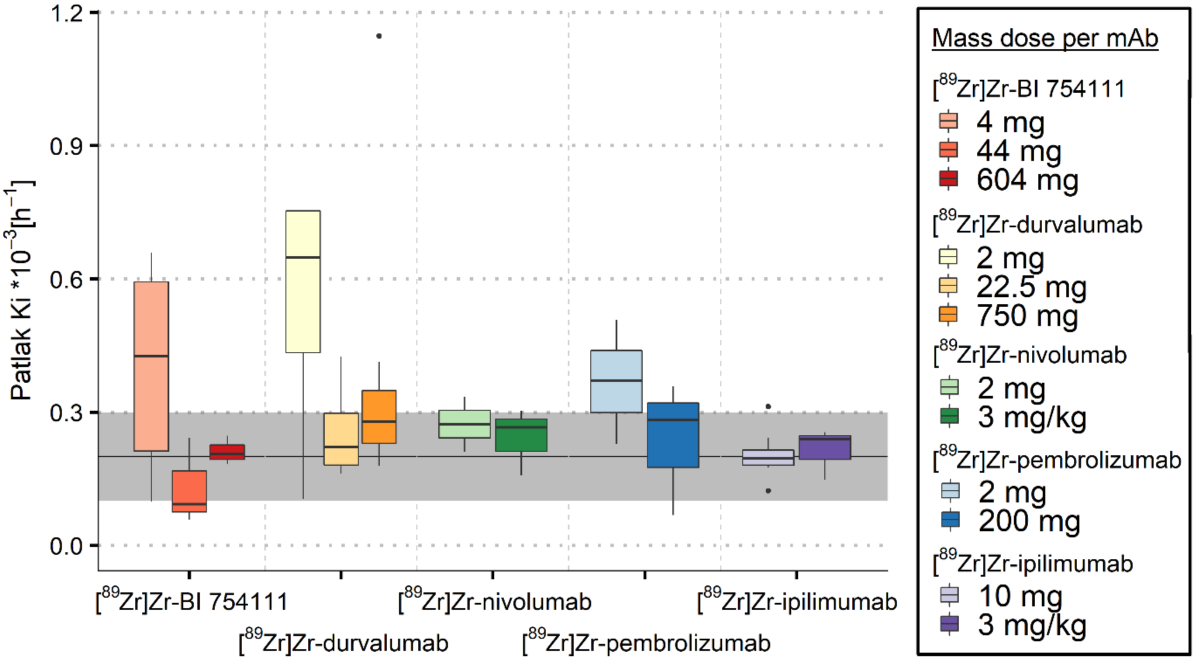

Figure S3. Irreversible uptake in lungs quantified with K_i values as a function of mass dose for the five [^{89}Zr]Zr-mAbs investigated. Median ns-baseline K_i values and interquartile range for lungs are indicated by the black line and grey area, respectively. K_i values decreased with increasing mass dose for three of five [^{89}Zr]Zr-mAbs. K_i values were comparable to ns-baseline values for all mass doses, except for the low mass doses of [^{89}Zr]Zr-BI 754111, [^{89}Zr]Zr-durvalumab and [^{89}Zr]Zr-pembrolizumab.
